# German guideline for the management of adverse reactions to ingested histamine: Guideline of the German Society for Allergology and Clinical Immunology (DGAKI), the German Society for Pediatric Allergology and Environmental Medicine (GPA), the German Association of Allergologists (AeDA), and the Swiss Society for Allergology and Immunology (SGAI)

**DOI:** 10.1007/s40629-017-0011-5

**Published:** 2017-02-27

**Authors:** Imke Reese, Barbara Ballmer-Weber, Kirsten Beyer, Thomas Fuchs, Jörg Kleine-Tebbe, Ludger Klimek, Ute Lepp, Bodo Niggemann, Joachim Saloga, Christiane Schäfer, Thomas Werfel, Torsten Zuberbier, Margitta Worm

**Affiliations:** 1 Medical Nutrition Therapy, Munich, Germany; 2 Allergy Ward, Department of Dermatology, Zurich University Hospital, Zurich, Switzerland; 3 Department of Pediatric Pneumology and Immunology, Charité University Hospital, Virchow Clinic Campus, Berlin, Germany; 4 Dermatology, Venereology, and Allergology Unit, Department of Dermatology, Göttingen University Hospital, Göttingen, Germany; 5 Westend Allergy and Asthma Center, Berlin, Germany; 6 Center for Rhinology and Allergology, Wiesbaden, Germany; 7 Practice for Respiratory Medicine and Allergology, Stade, Germany; 8 Department of Dermatology, University Hospital of the Johannes Gutenberg University, Mainz, Germany; 9 Specialist Allergy Practice, Hamburg, Germany; 10 Department of Dermatology, Allergology, and Venereology, Hannover Medical University, Hannover, Germany; 11 Department of Dermatology, Venereology, and Allergology Charité University Hospital, Charité Mitte Campus, Charitéplatz 1, 10117 Berlin, Germany

**Keywords:** Food, Adverse reaction, Histamine intolerance, Diamine oxidase, Stool analysis

## Abstract

Adverse food reactions are far more often perceived than objectively verified. In our scientific knowledge on non-allergic adverse reactions including the so called histamine intolerance, there are large deficits. Due to the fact that this disorder is increasingly discussed in the media and the internet, more and more people suspect it to be the trigger of their symptoms. The scientific evidence to support the postulated link between ingestion of histamine and adverse reactions is limited, and a reliable laboratory test for objective diagnosis is lacking. This position paper by the “Food Allergy” Working Group of the German Society for Allergology and Clinical Immunology (DGAKI) in collaboration with the German Association of Allergologists (AeDA), the Society for Pediatric Allergology and Environmental Medicine (GPA), and the Swiss Society for Allergology and Immunology (SGAI) reviews the data on the clinical picture of adverse reactions to ingested histamine, summarizes important aspects and their consequences, and proposes a practical diagnostic and therapeutic approach.

## Background and objectives of the guideline

Adverse reactions are more often subjectively perceived than objectively verifiable. There are large deficits particularly in our scientific knowledge on non-allergic adverse reactions. Histamine intolerance is an example in point: due to the fact that it is increasingly discussed in the media and Internet, subjectively affected individuals often suspect it to be the trigger of their symptoms. The scientific evidence to support this postulated link is limited, and a reliable laboratory test for the purpose of conclusive diagnosis is lacking. Although scientific studies on adverse reactions to ingested histamine have been carried out predominantly in adults to date, the diagnosis is also made in children and adolescents, often with significant consequences to the diets of affected individuals. Two reports on digestive disorders in children caused by histamine are now available [1, 2]. A retrospective observational study conducted in Spain [1] discusses histamine as a possible trigger; however, since diagnosis was based on low diamine oxidase (DAO) levels and dietary modifications without follow-up challenge, this study was not in line with recommendations. A German study [2] concluded that, although 50% of patients with suspected histamine intolerance responded to dietary changes, double-blind, placebo-controlled provocation was able to prove only one case of histamine intolerance.

This position paper by the “Food Allergy” Working Group of the German Society for Allergology and Clinical Immunology (DGAKI) in collaboration with the German Association of Allergologists (AeDA), the Society for Pediatric Allergology and Environmental Medicine (GPA), and the Swiss Society for Allergology and Immunology (SGAI) reviews the data on the clinical picture of adverse reactions to ingested histamine, summarizes important aspects and their consequences, and proposes a practical diagnostic and therapeutic approach.

Ingested histamine is often suspected as the cause of (unspecific) symptoms, despite the fact that the scientific data supporting a clinical picture of this type is limited.

## Occurrence, function, and degradation pathways of histamine

**Fig. 1:**
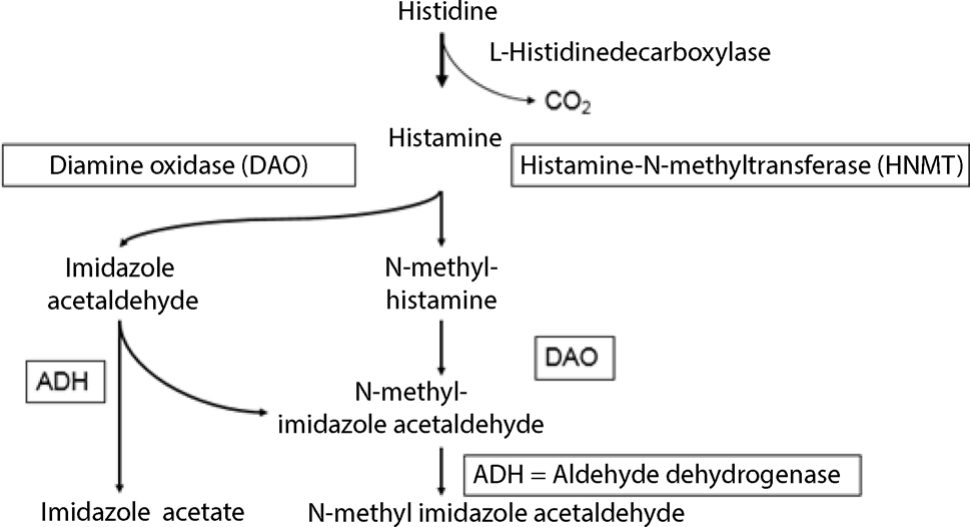
Histamine degradation pathways

Histamine is a biogenic amine derived from the decarboxylation of the amino acid histidine. Endogenously synthesized histamine, which is stored primarily in mast cells and basophils, is one of the most important mediators not only of IgE-, but also of non-IgE-dependent clinical reactions. The body metabolizes histamine via two known degradation pathways (Fig. 1):

- Methylation by histamine-N-methyltransferase (HNMT),
- Oxidative degradation by diamine oxidase (DAO).

Ingested histamine also needs to be metabolized via these degradation pathways. Large quantities can cause poisoning: histamine quantities above 100 milligrams (mg) can elicit mild, quantities above 1000 mg severe intoxication. Histamine intoxication most commonly occurs following the consumption of spoiled fish, particularly from the family Scombridae (tuna, mackerel, etc.).

## Diagnostic work-up for adverse reactions to ingested histamine

There is no reliable procedure as yet for the diagnosis of adverse reactions to ingested histamine. Since it is most often self-diagnosed by the patient or solely based on patient history, it is helpful to examine relevant aspects more closely in order to answer the following questions: Which symptoms can be expected and which differential diagnosis could be helpful? Is there evidence of the presumed pathomechanism? Which parameters are described for the diagnostic work-up and how reliable are they? Can drug therapy influence the disease pattern? The confirmation of the suspected diagnosis by oral provocation is also discussed, as are the difficulties that arise due to variable levels in foods. An explanation of terms rounds up the data discussed here.

### Symptoms and differential diagnoses in patients with suspected adverse reactions to ingested histamine

The spectrum of symptoms in the so called “histamine intolerance” can affect several organ systems and is complex. Classic symptoms include sudden facial erythema (flushing), pruritus, and rashes on the body. Histamine can also cause gastrointestinal symptoms, such as nausea and/or vomiting/diarrhea and abdominal pain. Symptoms related to the respiratory tract, as well as cardiovascular symptoms such as low blood pressure, dizziness, or tachycardia, are rarer but not unknown [3–5].

Given the extensiveness of clinical symptoms, it is important to make a broad differential diagnosis covering skin disorders such as urticaria, as well as chronic inflammatory gastrointestinal disorders, carbohydrate metabolism disorders, celiac disease, and other allergic diseases (Table 1).

**Table 1:** Symptoms and differential diagnoses in patients with suspected adverse reactions to ingested histamine

Symptoms	Differential diagnosis(es)
Flushing^a^	Neuroendocrine tumors
Itching^a^	Urticaria, pruritus sine materia, prurigo
Nausea/vomiting^a^	Gastric ulcer, duodenal ulcer
Diarrhea and abdominal pain^a^	Chronic inflammatory bowel disorders, carbohydrate metabolism disorders (lactose intolerance, fructose malabsorption), celiac disease
Rhinitis^a^	Allergic and non-allergic rhinitis
Dyspnea, dysphonia^a^	Allergic and non-allergic asthma
Low blood pressure, dizziness, tachycardia^a^	Anaphylaxis

^a^Analyzing symptoms with reference to their temporal onset provides important differential diagnostic information; adverse food reactions are only suspected in the case of a temporal relationship (min <4 h) to food intake

### Presumed pathomechanism

Since the mid 1980s, ingested biogenic amines have been suspected of causing adverse reactions in some individuals even in small quantities, which are below the toxic dose. The discussion has focussed on adverse reactions elicited by histamine-containing foods, despite the fact that other biogenic amines (cadaverine, tryptamine, tyramine, serotonin, etc.) and/or polyamines (putrescine, spermine, spermidine, among others) also cause adverse reactions or may affect histamine metabolism [6]. The suspected pathomechanism for reactions to ingested histamine involves impaired degradation by catabolizing enzymes, primarily DAO [4]. The term “histamine intolerance” was coined with reference to the term “lactose intolerance” (due to enzyme deficiency). However, prospective controlled studies conclusively demonstrating that an enzyme and/or deficient enzyme activity is/are the cause of reactions to ingested histamine are lacking to date. It must be borne in mind that histamine can be degraded via two pathways (Fig. 1).

Impaired histamine catabolism due to diamine oxidase deficiency is postulated as the cause of adverse reactions to ingested histamine; this causal link, however, has yet to be proven.

### The described diagnostic parameters and their reliability

A number of different parameters have been proposed for the diagnosis of “histamine intolerance”; these are discussed below with regard to their reliability.

#### DAO activity in serum

According to current data, diagnosis based on the measurement of DAO enzyme activity in blood serum cannot be considered conclusive [7, 8]. Using DAO-specific monoclonal antibodies, it was possible to detect DAO in various tissues, such as kidneys, intestine, and placenta—not, however, in blood serum, or at least not in relevant quantities [9]. This finding casts conclusive doubt on the utility of serum analysis.

Nevertheless, a recent study has once again proclaimed the reliability of determining DAO activity in blood serum [10]. In stark contrast to earlier works [7, 8], DAO values in that particular study’s limited control group were in the normal range.

#### Histamine 50-skin-prick test

Meanwhile, Kofler and coworkers have published a study that could indeed support slowed histamine degradation [11]. As part of the so-called histamine 50-prick-test, the histamine wheal was re-read after 50 min. If wheal size remained unaltered up to that time, impaired degradation was assumed. However, this method does not permit conclusions to be drawn on whether the degradation of ingested histamine is slowed down and needs to be viewed highly critically.

#### Measurement of intestinal enzyme activity

Potential diagnostic significance is attributed to the measurement of enzyme activity (or activities) (DAO and possibly HNMT) in the intestinal mucosa, since this is considered the most important organ for the degradation of histamine from exogenous sources. However, according to current knowledge, blood DAO levels in humans—in contrast to animals—do not permit any conclusions to be drawn on enzyme activity of DAO in the small intestine [12].

Further scientific investigation is needed to establish whether the determination of DAO activity in the small intestinal mucosa yields information on the capacity to degrade exogenous histamine.

Kuefner and coworkers demonstrated a trend toward reduced DAO activity in the colonic mucosa of patients with food allergies, but without reaching significance levels [13]. HNMT activity, on the other hand, was markedly diminished; parallel to this, histamine values in the intestinal mucosa were elevated. The authors see this diminished HNMT activity as the primary cause of impaired histamine metabolism in the colon. However, the study did not investigate the effect of ingested histamine.

The same working group showed that not only DAO activity, but also to a greater extent HNMT activity was likewise diminished in damaged tissue in colonic adenoma patients [14]. The authors found slightly increased histamine concentrations in bowel tissue; however, this did not correlate with enzyme activity. They concluded that the histamine in the colonic mucosa was more likely to be elevated due to increased release rather than reduced degradation. The effect of exogenous histamine intake was not investigated.

Histamine in the intestine is degraded not only by diamine oxidase, but possibly also by histamine N‑methyl-transferase.

#### Histamine in stool samples

It is now known that some bacteria of the intestinal microbiota, in particular lactobacilli, partially secrete large quantities of histamine. This casts doubt on the validity of high histamine levels in stool samples as pathological. O’Mahony and his working group showed in a mouse model that histamine produced in the intestinal lumen—depending on which histamine receptor (HR) it binds to—not only has proinflammatory, but also regulatory effects on the immune system. If the secreted histamine binds to the histamine H2 receptor (H2R), it has more of a regulatory effect [15–17].

The fact that histamine is a relevant metabolite of intestinal bacteria casts doubt on the validity of diagnostic stool analysis.

#### Histamine levels in plasma

The significance of determining plasma histamine levels is subject to scientific controversy. Giera and coworkers performed provocation tests in patients with suspected histamine intolerance and controls with 75 mg histamine and placebo [18]. The rise in plasma histamine following verum administration was minimal in patients with suspected histamine intolerance and did not differ from the control group, not even in those patients that exhibited symptoms in response to provocation. In the control group, on the other hand, a marked rise in plasma histamine was observed following verum administration, albeit without accompanying symptoms.

#### Methylhistamine in urine

The determination of methylhistamine in urine needs to be critically examined, since methylhistamine levels depend not only on histamine, but also in general on the protein content of foods and also rise on a high-protein but low-histamine diet [19].

There are no objective parameters as yet for the presence of adverse reactions to ingested histamine.

### Relevance of medications

A number of medications have been deemed to have a negative effect on histamine-degrading enzymes, primarily DAO [20, 21]. Medications such as acetylcysteine, metamizole, verapamil, metronidazole, and metoclopramide have been mentioned [22, 23]. According to a recent literature search, data on these older reports is inconsistent. More research is needed to validate the effect of these and other medications on histamine-degrading enzymes and to identify potential pharmacological interactions in exogenously absorbed histamine.

The relevance of particular medications with regard to the degradation capacity of diamine oxidase needs to be validated in further studies.

### Oral provocation with histamine: between a diagnostic threshold dose and inadvertent poisoning

The best method to verify an adverse food reaction is titrated oral provocation, ideally performed in a double-blind, placebo-controlled test design that should have clinical parameters as endpoints. As yet, there is no established procedure that can be applied in routine practice for suspected adverse reactions to ingested histamine. The specification of an appropriate provocation dose is a prerequisite of diagnostically valid oral provocation. The ideal dose should fail to elicit a reaction in a sufficiently large collective of healthy subjects, while inducing the described symptoms in subjects suspected to be intolerant. If unexpected systemic reactions not described in the patient history are observed, the dose was too high.

The 75-mg dose, which is most frequently selected in the studies to date, triggered symptoms in 50% of healthy subjects in one particular study [5]. In an analysis of patients with atopic dermatitis (AD), systemic reactions were seen in seven patients and four control subjects following administration of a 75-mg dose of histamine dihydrochloride (1 mg histamine is equivalent to 1.6 mg histamine dihydrochloride) per kilogram body weight (mg/kg BW). In another study, administration of a dose of 1.5 mg/kg BW elicited reactions in 14 AD patients and 11 control subjects [24].

The fact that, in both studies, the provocation doses triggered reactions in healthy controls prompts the suspicion of subtoxic effects. It is questionable, therefore, whether these doses are suited for diagnostic purposes in suspected adverse reaction to ingested histamine.

#### Recommended approach

**Fig. 2:**
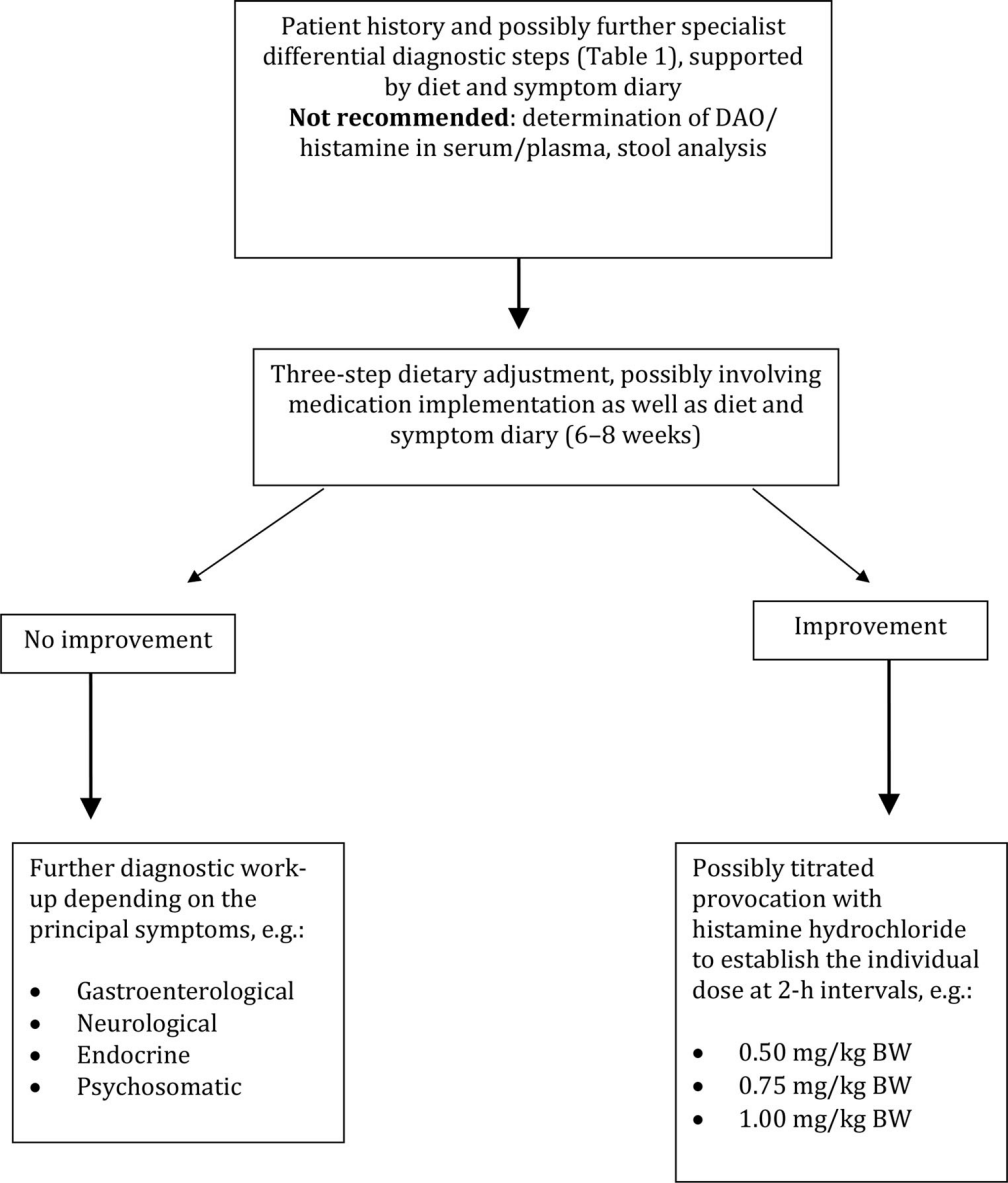
Proposed approach in patients with suspected adverse reactions to ingested histamine

Until a validated test system is available, the following approaches are proposed for the identification of symptoms caused by histamine intake (Fig. 2): In a first step, patients should undergo a three-step dietary adjustment (Table 2). Titrated provocation with ascending doses of histamine hydrochloride at 2‑h intervals (e. g., 0.5 mg/kg BW, 0.75 mg/kg BW, up to 1.0 mg/kg BW) could then be considered to determine the individual’s tolerated dose. Titrated histamine provocation should be performed under medical supervision, since systemic reactions ranging from nausea and vomiting to temporary circulatory dysregulation may occur. These symptoms are generally transient and can be managed by administration of antihistamine agents.

**Table 2:** Phases of the three-step dietary adjustment

Phase	Aim	Recommendation	Duration
Phase 1: avoidance	To reduce symptoms to the greatest possible extent	– Mixed diet with emphasis on vegetables and reduced biogenic amine intake, in particular histamine intake – Nutrient optimization – Changes in meal composition – Principles of a balanced diet	10–14 Days
Phase 2: test phase	To expand the choice of food while taking individual risk factors (stress, menstruation, medication use, etc.) into account	– Targeted re-introduction of suspected foods while taking the patient’s individual dietary preferences into consideration – Determination of individual histamine tolerance	Up to 6 weeks
Phase 3: long-term diet	Continuous, balanced supply of nutrients High quality of life	– Individual nutritional recommendations guided by the individual histamine tolerance, taking exogenous risk factors into consideration	–

#### Factors influencing the provocation result

When performing oral provocation tests it must be borne in mind that individual sensitivity is subject to considerable variability and numerous concomitant factors affect intestinal permeability, including the following:

- The use of acetylsalicylic acid, other non-steroidal anti-inflammatory drugs, as well as other medications,
- Various, but primarily inflammatory, intestinal disorders,
- Concomitant alcohol use,
- Hormone status,
- Probably also the composition of intestinal flora and other factors.

Due to a lack of data, it is currently not possible to propose a suitable histamine dose or an appropriate procedure for performing oral provocation testing. In the meantime, titrated provocation tests using estimated individual tolerated doses can yield information on the possible presence of impaired histamine metabolization.

### Variable histamine content in foods

The diagnosis and treatment of adverse reactions to ingested histamine is hampered by the fact that the histamine content in food varies significantly depending on maturity, storage time, and processing. As a result, histamine levels can differ considerably within the same food product. For example, the histamine content in Emmental cheese varies from <0.1 to 2000 mg/kg and in smoked mackerel from <0.1 to 1788 mg/kg [25]. These variations make it difficult to estimate the histamine content of individual meals.

Furthermore, some of the dietary recommendations that are currently circulating are not supported by scientific evidence. For example, numerous low-histamine diets prohibit foods that do not contain histamine (e. g., yeast), or encourage the avoidance of so-called “histamine liberators” (pharmacologically active substances that have a histamine-releasing effect), despite there being no reliable evidence of their existence in foods or of their clinical relevance in the onset of adverse food reactions [26]. The inconsistent data on biogenic amines in foods make it difficult to issue safe recommendations on diagnosis and define treatment measures.

Histamine levels in foods are subject to significant variation—even within the same food product—depending on maturity, storage time, and particular treatment processes, thereby hampering diagnosis and medical guidance.

### Explanation of terms: adverse reactions to histamine or histamine intolerance

In summary, it is currently not established whether an individual sensitivity to ingested histamine is due to an enzyme defect. The so-called histamine intolerance is more likely a “complex of symptoms that can be attributed to histamine only in individual cases” than an isolated clinical picture exclusively triggered by ingested histamine [27]. That would explain why it is often not possible to reproduce symptoms following small quantities of histamine in the patient history [27, 28]. It is possible that symptoms only appear in the presence of concomitant factors (see above). In this context, an alteration in small intestinal permeability as a prerequisite of symptom onset may be of relevance. However, the study conducted by Komericki and coworkers [27, 28] raises the question of whether symptoms can be affected by orally administered histamine at all. The intake of the histamine-degrading enzyme DAO was described to reduce symptoms irrespective of whether or not histamine was administered [28].

We propose favoring the term adverse reaction to ingested histamine until the underlying pathomechanism for reactions to minute quantities of histamine, i. e., far below the toxicologically relevant dose, has been elucidated.

A clarification of the differences between the terms adverse reaction to ingested histamine and histamine intolerance is still pending.

## Practical approach in daily routine

Even without knowing the underlying pathomechanism, patients with suspected an adverse reaction to ingested histamine can receive helpful advice on how to alter their dietary habits. In a first step, however, possible differential diagnoses (see section “Symptoms and differential diagnoses in patients with suspected adverse reactions to ingested histamine”) should be investigated and, where necessary, treated. If the suspicion of an adverse reaction to ingested histamine persists, individually relevant quantities of biogenic amines as well as concomitant factors that either induce or promote adverse reactions can be best identified by using a symptom and food diary.

There are several factors capable of increasing the sensitivity to histamine (see section “Factors influencing the provocation result**”**). An increase in symptoms is observed in female patients during the premenstrual phase [4]. But dietary factors, such as food selection, meal composition, and intervals between meals, can also affect symptoms.

### Dietary adjustment

Experience to date (e. g., in the context of individualized nutritional therapy) has shown that tolerance to histamine and biogenic amines can be increased by a three-step dietary adjustment (Table 2). However, controlled studies are needed to investigate to what extent nutritional changes are actually able to achieve biological effects and to affect the natural course of tolerance. On the other hand controlled studies could shed light on how strong psychological effects are achievable by expert advice.

Taking our limited knowledge on the pathomechanism of adverse reactions to ingested histamine into consideration, the approach described here (Fig. 2) is recommended in order to avoid generalized, restrictive, and long-term low-histamine diets that unnecessarily reduce patients’ quality of life.

A diagnostic work-up, combined with individualized nutritional therapy that focuses primarily on nutrient optimization and helps patients reliably to differentiate symptoms, is to be preferred over generalized, restrictive diets.

### The value of antihistamine agents

There are no double-blind, placebo-controlled prospective studies on the efficacy of H1 and H2 receptor blockers in patients with adverse reactions to ingested histamine. However, the mode of action of these drugs suggests that they ought to work in the treatment of individual symptoms (e. g., H1 blockers for flushing and H2 blockers for nausea/vomiting)—at least in the acute setting (severe dietary errors, e. g., in the context of festive meals, or scombroid poisoning) [29–31].

In a pragmatic approach, one could conceivably treat patients with suspected adverse reactions to ingested histamine with H1/H2 receptor blockers for a certain period of time in order to investigate whether this alters symptoms.

## Conclusion and outlook

In summary, the points discussed here show that the diagnosis of adverse reactions to ingested histamine has hitherto been made purely on the basis of symptoms and in the absence of reliable laboratory parameters. The treatment approach should be largely guided by the individual tolerance of affected individuals. Generalized restrictions on food selection are only relevant for diagnostic purposes and do not help affected patients in the long term.

More research is needed to establish the relevance of measuring biomarkers, risk factors in intestinal function and barrier, as well as the histamine dose that elicit pharmacological effects of histamine. Until then, expert nutritional counseling can help patients to avoid diets that result in an unnecessary reduction in their quality of life.

### Consensus procedure.

Guideline by informal consensus by experts of all participating scientific medical societies after search for relevant studies

### Facilitator.

Prof. Dr. med. Margitta Worm, Berlin

### Development level.

S1

### AWMF guideline register number.

061/030

### ICD-10 number.

T78., T61., K90.4
